# A Case of Gastric Diverticulum Presenting As Upper Gastrointestinal Bleeding in Rural Central India

**DOI:** 10.7759/cureus.28403

**Published:** 2022-08-25

**Authors:** Satish Mahajan, Srinivas Naik, Anamika Giri, Gaurav S Jagtap, Sourya Acharya

**Affiliations:** 1 Medicine, Jawaharlal Nehru Medical College, Datta Meghe Institute of Medical Science (Deemed to be University), Wardha, IND

**Keywords:** endoscopy, varices, bleeding, gastric, diverticulum

## Abstract

Gastric diverticula (GD) are the least prevalent type of gastrointestinal diverticula and are extremely uncommon anatomical anomalies in general. Although the majority of GD cases are asymptomatic and are identified by chance during normal diagnostic testing, they can manifest with a variety of symptoms. In some instances they can lead to life-threatening problems, necessitating surgical intervention. The majority of gastric diverticula go unnoticed. Upper abdomen pain, nausea, emesis, and dyspepsia are the most prevalent symptoms. Patients with GD can present with dramatic symptoms such as major bleeding or perforation on rare occasions. We report a rare case of a 60-year-old male, who presented with a complaint of haematemesis, and upon doing endoscopy and contrast-enhanced computed tomography of the abdomen was diagnosed with GD. The patient was managed successfully with proton pump inhibitor infusion and somatostatin analogs and was discharged in stable condition. Here, we highlight a rare but potentially life-threatening cause of hematemesis which is often missed by treating clinicians, especially in rural and remote areas, and therefore, requires more awareness and clinical vigilance.

## Introduction

Gastric diverticula (GD) are outpouchings of the stomach wall that most commonly occur in the fundus and along the posterior wall. They have characteristics that are similar to the small bowel and colonic diverticula [1,2]. Patients with GD may experience vague epigastric pain or fullness, bleeding, or symptoms of perforation. Gastric diverticula are typically 1 cm to 3 cm in diameter and are classified as true diverticula, which includes all gastrointestinal layers, and pseudodiverticula, which are frequently detected in the antrum. Seventy-Five percent of true gastric diverticula are found in the fundus of the stomach's posterior wall, 2 cm below the gastroesophageal junction and 3 cm from the lesser curvature [3]. False diverticula are connected with inflammation, other disorders, or both, and are formed by either traction or pulsion. The size of the diverticula is usually less than 4 cm.

Before fusing with the left posterior body wall, the gastric fundus' posterior wall might conceivably herniate through an area of dorsal mesentery. The diverticulum would be higher than the pancreas at first. The diverticulum may be able to project posteriorly to the pancreas if it is extended further. Pseudodiverticula, on the other hand, are acquired gastric diverticula that are less prevalent and usually found in the antrum [3]. They frequently have a history of peptic ulcer disease, cancer, pancreatitis, or gastric outlet obstruction. Gastric diverticula have been documented following stomach surgery methods such as the Roux-en-Y gastric bypass. We report a case of an older adult who presented with the chief complaint of hematemesis, which upon investigation turned out to be a case of gastric diverticulum.

## Case presentation

A 60-year-old male patient came to the emergency department with the chief complaint of blood in vomitus for five days, approximately 50 ml every episode, reddish-brown in color, with around one to two episodes per day since. The patient is a non-alcoholic and a non-smoker.

On the first day of admission, the patient was drowsy and in shock with a blood pressure of 80/50 mmHg, pulse rate at 124 beats per minute, respiratory rate of 30 cycles per minute, and pallor present. On systemic examination, the abdomen was soft and non-tender, respiratory system examination revealed a clear chest, and cardiovascular system revealed normal heart sounds with no murmur. The patient's coagulation profile, kidney function tests, and liver function tests were normal. However, stool for occult blood was positive. As the patient was severely anemic, he was transfused with four units of packed red cells. Serial monitoring of complete blood count was done (Table 1). He was treated with pantoprazole and octreotide infusion, fluid resuscitation, and ionotropic support (noradrenaline).

**Table 1:** Report of complete blood investigation

Investigation & date	14/01/2022	14/01/2022	15/01/2022	16/01/2022	17/01/2022	19/01/2022
Hemoglobin gm%	5.2	7.8	7.4	7.8	9.6	10.9
White Blood Cells/cumm	12300	12600	9400	6300	7700	9900
Platelet count lakhs/cumm	3.36	3.67	3.22	3	3.24	5.62

**Figure 1:**
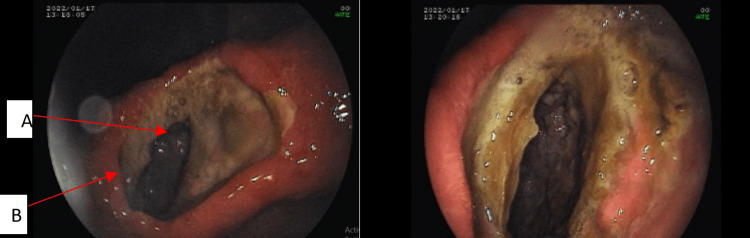
Diverticular opening with large necrotic slough and an ulcer is seen A: Large necrotic slough, B: Diverticula border with ulcer

**Figure 2:**
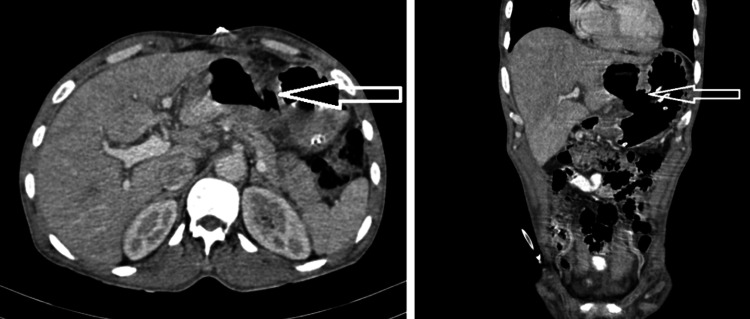
Axial and coronal contrast-enhanced CT shows a well-defined saccular outpouching noted at the lesser curvature stomach (at the body) filled with oral contrast and seen communicating (neck size: 2.8 cm) with the gastric cavity.

An upper gastrointestinal endoscopy (Figure 1) was performed but was inconclusive for the cause of hematemesis since a lot of blood was present in the stomach. To ascertain the cause of hematemesis a contrast-enhanced CT abdomen was done which showed a diverticulum in the lesser curvature of the stomach (at the body) (Figure 2).

Having been diagnosed with gastric diverticulum that presented with significant bleeding leading to hypovolemic shock and severe anemia, the patient was advised surgical management in the form of resection of the diverticulum. As the patient improved symptomatically and was unwilling to undergo surgery, he was discharged on proton pump inhibitors, oral iron, folic acid, and vitamin B12 .

## Discussion

True diverticula and false diverticula are two forms of GD, with congenital diverticula being more prevalent. All layers of the stomach wall are present in true diverticula. False diverticula, on the other hand, do not feature all the layers. Furthermore, there are two types of false diverticula: pulsion and traction. Increased intraluminal pressure caused by chronic coughing or obesity, causes pulsion diverticula. Conversely, traction diverticula develop as a result of contractile forces i.e., either from an adjacent inflammatory process or from peri gastric adhesions from coexisting illnesses. These include peptic ulcer disease, pancreatitis, cholecystitis, cancer, gastric outlet obstruction, and gastroesophageal reflux disease. In particular, traction diverticula in the stomach have been reported to occur following surgical procedures, including Roux-en-Y gastric bypass surgery [4].

Making an accurate diagnosis in cases with GD is critical due to the risk of rare but serious consequences, as well as the link to ectopic mucosa and the chance of a malignant conversion. Gastric cancer has been reported in and near GD, which is particularly worrying. As a result, a thorough endoscopic evaluation and biopsies from GD with questionable, elevated, or irregular borders are recommended [5]. The majority of the time, GD is detected by chance during normal diagnostic testing for typical gastrointestinal symptoms. However, the procedures employed to detect them may fail, necessitating the employment of a combined diagnostic strategy. Radiological studies (upper gastrointestinal contrast radiography study and abdominal CT scanning with oral contrast administration) and upper gastrointestinal endoscopic examinations can both be used to diagnose GD.

The severity of the presenting problems, as well as the size of the diverticulum, play a big role in GD treatment. Asymptomatic persons, as is well established, do not require therapy and can be left alone. The majority of diverticula are congenital, discovered by chance, and asymptomatic, thus they don't need to be treated. Diverticula that cause substantial symptoms or difficulties, and that are usually large, should be resected because there is no alternative viable treatment [6].

Gastric diverticulum is one of the rarest causes of hematemesis. A patient with GD presenting with hematemesis is one of the indications for surgery. In our patient, the symptom of hematemesis initiated an investigation which unleashed the diagnosis of gastric diverticulum. As our patient was 60 years old this blood loss in the form of hematemesis could have been life-threatening if not managed on time. A prompt diagnosis followed by management with proton pump inhibitors and somatostatin analogs helped in preventing mortality and morbidity. The patient recovered well and was discharged.

## Conclusions

Gastric diverticula are uncommon and necessitate a strong clinical index of suspicion on the part of the clinician. Their existence may be linked to vague symptoms including pain, discomfort, and dyspepsia. Other hygiene-related symptoms that have a significant social impact, such as halitosis, may prompt a GD examination. In the diagnosis of GD, the upper gastrointestinal (UGI) series and esophagogastroduodenoscopy (EGD) are crucial. When the GD is large, or when patients have not reacted well to medication treatment with the development of further problems, a laparoscopic resection supported by intraoperative endoscopy should be considered a safe and practical surgical treatment.
